# Supplementary material for: Phage endolysin enables targeted manipulation of the small intestinal microbiota and uncovers niche overlap between oral and butyrate-producing taxa
Source: NPJ Biofilms Microbiomes. 2026 Jul 13;12:223. doi: 10.1038/s41522-026-01087-4 (PMC13737061; doi:10.1038/s41522-026-01087-4)
Supplement: Supplementary file 1 [file 41522_2026_1087_MOESM1_ESM.pdf]

SUPPLEMENTARY DATA

Phage Endolysin Enables Targeted Manipulation of the Small Intestinal Microbiota and Uncovers Niche Overlap Between Oral and Butyrate-Producing Taxa

Julian R. Garneau[1,#,*], Aline Altenried[1,#], Youzheng Teo[1], Xiaobing Wu[1], Sarah McHugh[1], Michael Taschner[1], Carla Hernández-Cabanyero[1,2], Johann Mignolet[1], Pénélope Gomes[3], Hugues de Villiers de la Noue[3], Grégory Resch[3,+], Pascale Vonaesch[1,+,*]

[1] Department of Fundamental Microbiology, University of Lausanne, Lausanne, Switzerland.
[2] current address : Interactions Hôtes-Pathogènes-Environnements (IHPE), UMR 5244, CNRS, Université de Montpellier, Université de Perpignan, IFREMER, Montpellier, France.
[3] Laboratory of Bacteriophages and Phage Therapy, Center for Research and Innovation in Clinical Pharmaceutical Sciences (CRISP), Lausanne University Hospital, Lausanne, Switzerland.

[#] Co-first authors
[+] Co-last authors

* Corresponding authors: julian.garneau@unil.ch; pascale.vonaesch@unil.ch

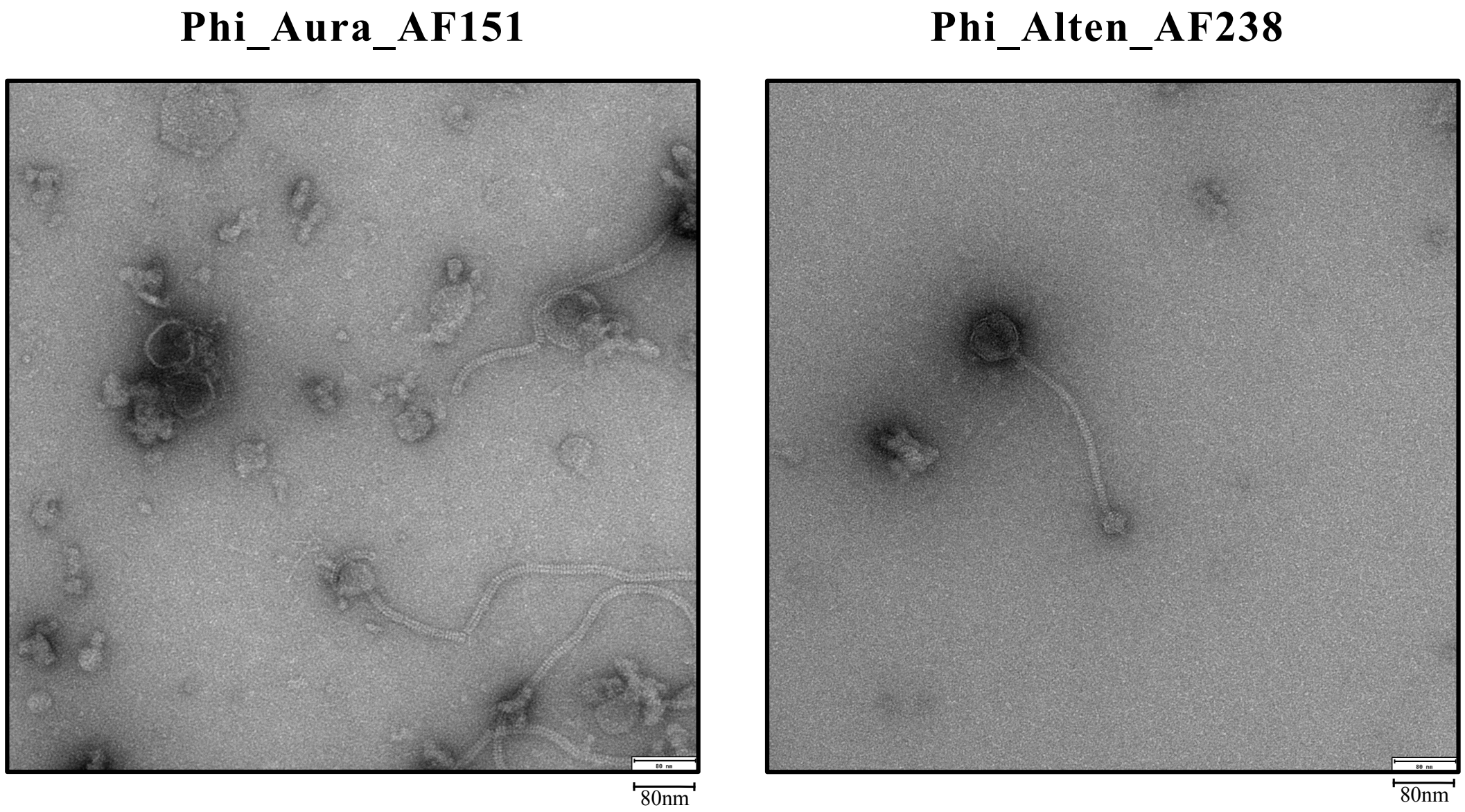

Supplementary Figure 1. Transmission electron microscopy (TEM) images of phages phi_Aura_AF151 and phi_Alten_AF238. Phages were induced from *S. salivarius* strains AF151 and AF238, respectively. Both phages display *Siphovirus* morphology, characterized by long, non-contractile tails.

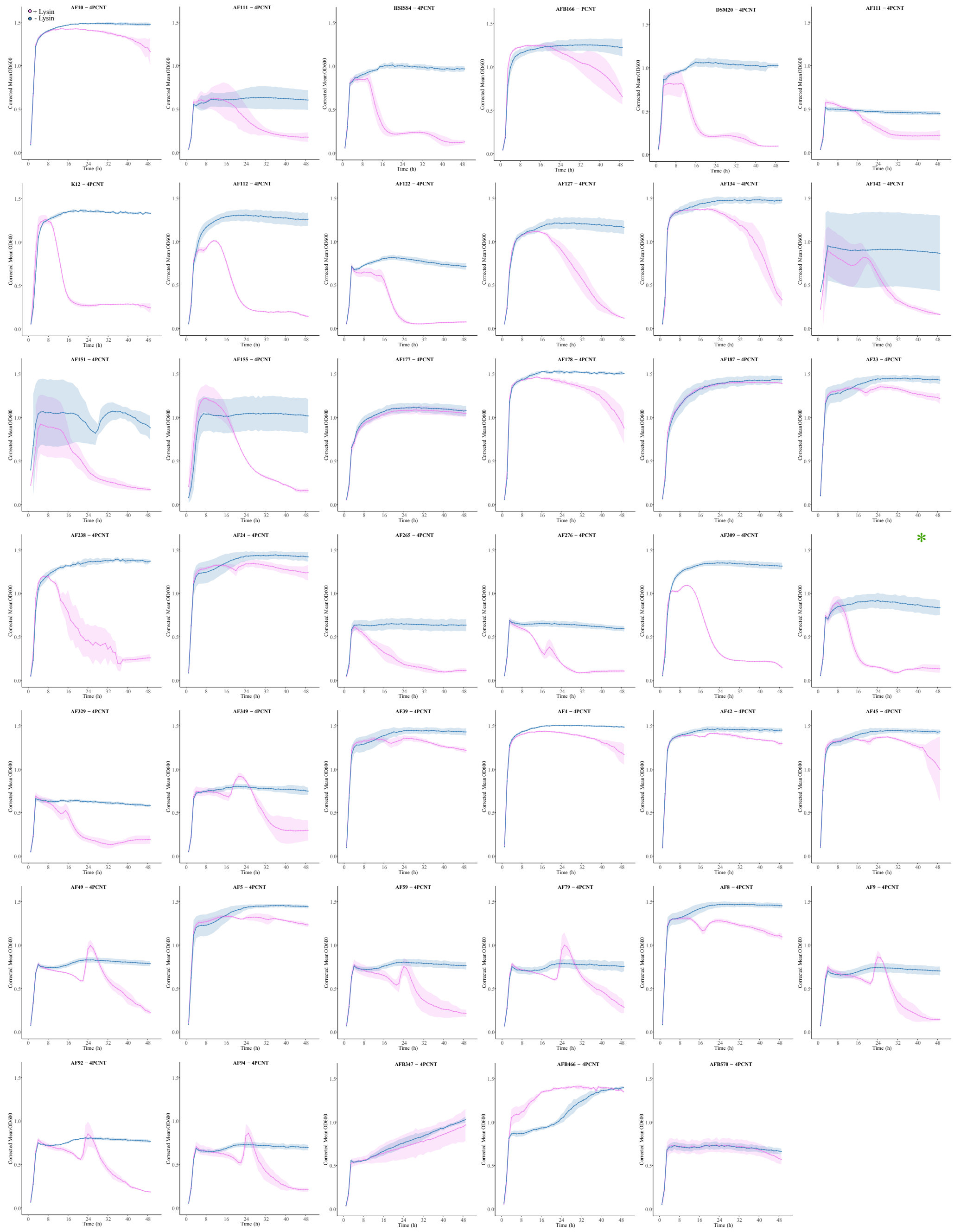

Supplementary Figure 2. Growth inhibition of *S. salivarius* isolates by Lys_AF151 lysin.
OD600nm growth curves over 48 hours for *S. salivarius* isolates grown in BHI medium with (Pink) or without (Blue) endolysin Lys_AF151 treatment. Each panel represents one isolate (strain name indicated above each plot). Growth was monitored every hour under anaerobic conditions. All experiments were performed in triplicates.

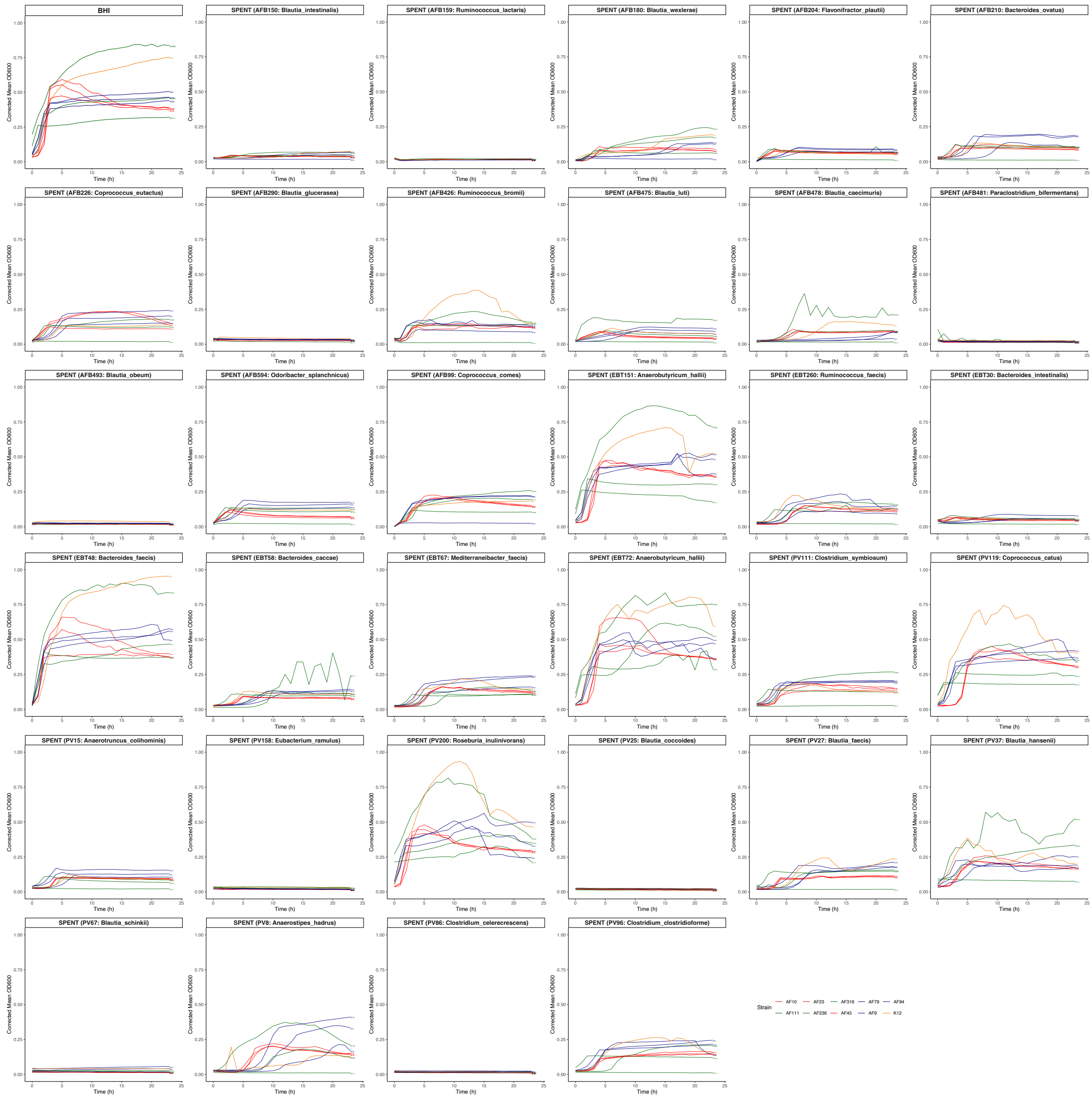

Supplementary Figure 3. Results of the spent medium assay. The 10 selected *S. salivarius* strains were grown in fresh BHI for reference growth curves and grown in the spent medium from 33 "donor strains". Each panel represents the growth in the spent medium of one specific donor strain. All experiments were performed twice with a single, representative experiment represented here. Colors for growth curves were assigned to *S. salivarius* strains according to the ANI cluster they belong (Red : cluster 1 = C1, Green : cluster 2 = C2, Blue : cluster 3 = C3, Orange : cluster 4 = C4

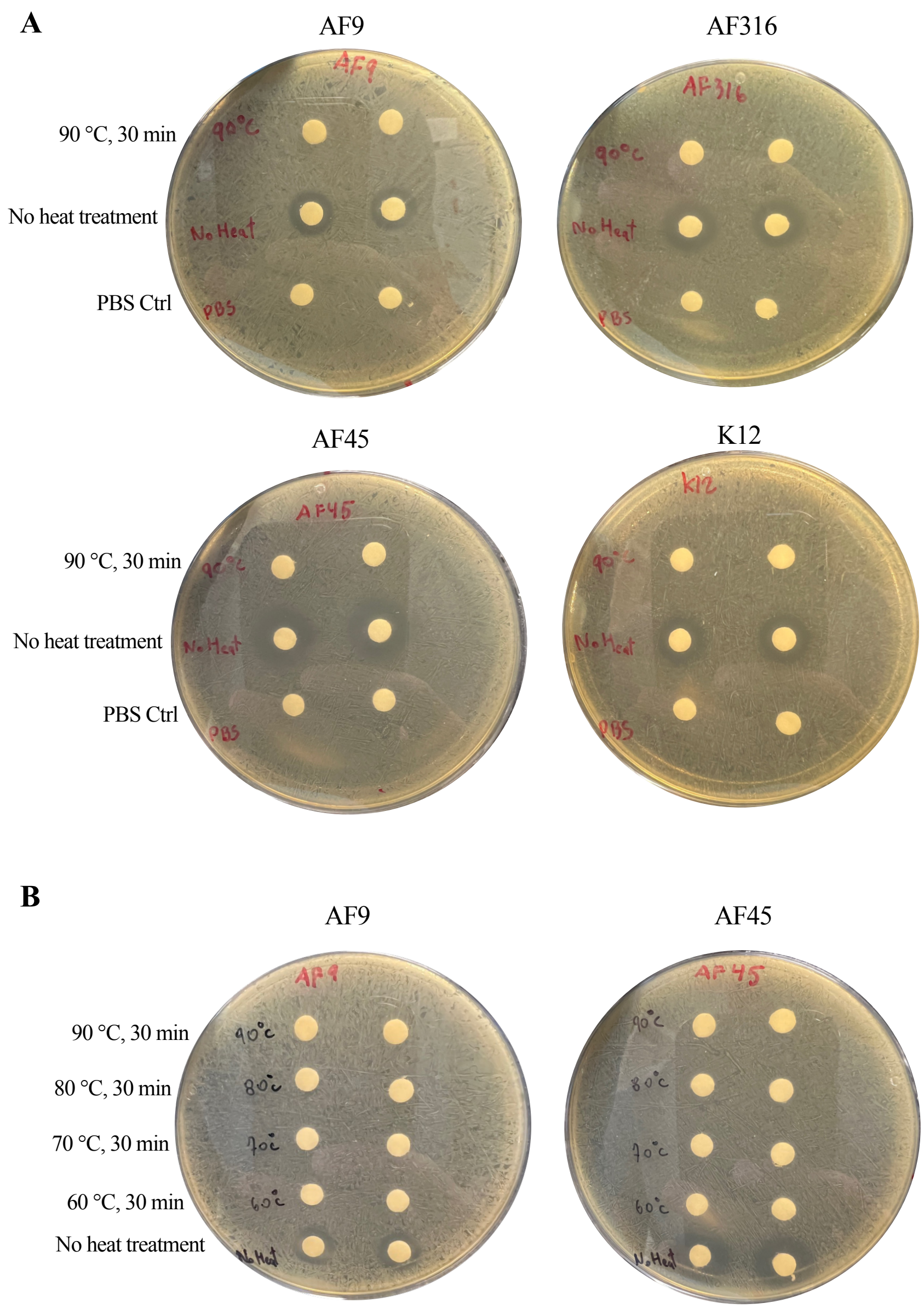

Supplementary Figure 4. Thermal stability of lysin antibacterial activity. Purified lysin was incubated at the indicated temperatures for 30 min or left untreated, and residual antibacterial activity was assessed by a spot-on-lawn assay. Twenty microlitres of lysin solution (0.05 mg/mL) were applied onto Whatman paper discs placed on bacterial lawns, with PBS included as a negative control. (A) Initial screen of lysin activity against AF9, AF45, AF316, and Escherichia coli K-12 after heat treatment at 90 °C for 30 min, compared with untreated lysin and PBS controls. (B) Temperature-range assay showing residual lysin activity against AF9 and AF45 after incubation at 60 °C, 70 °C, 80 °C, or 90 °C for 30 min, compared with untreated lysin and PBS controls. Plates were incubated at 37 °C for 24 h, and lysin activity was evaluated visually based on the presence of growth inhibition zones.

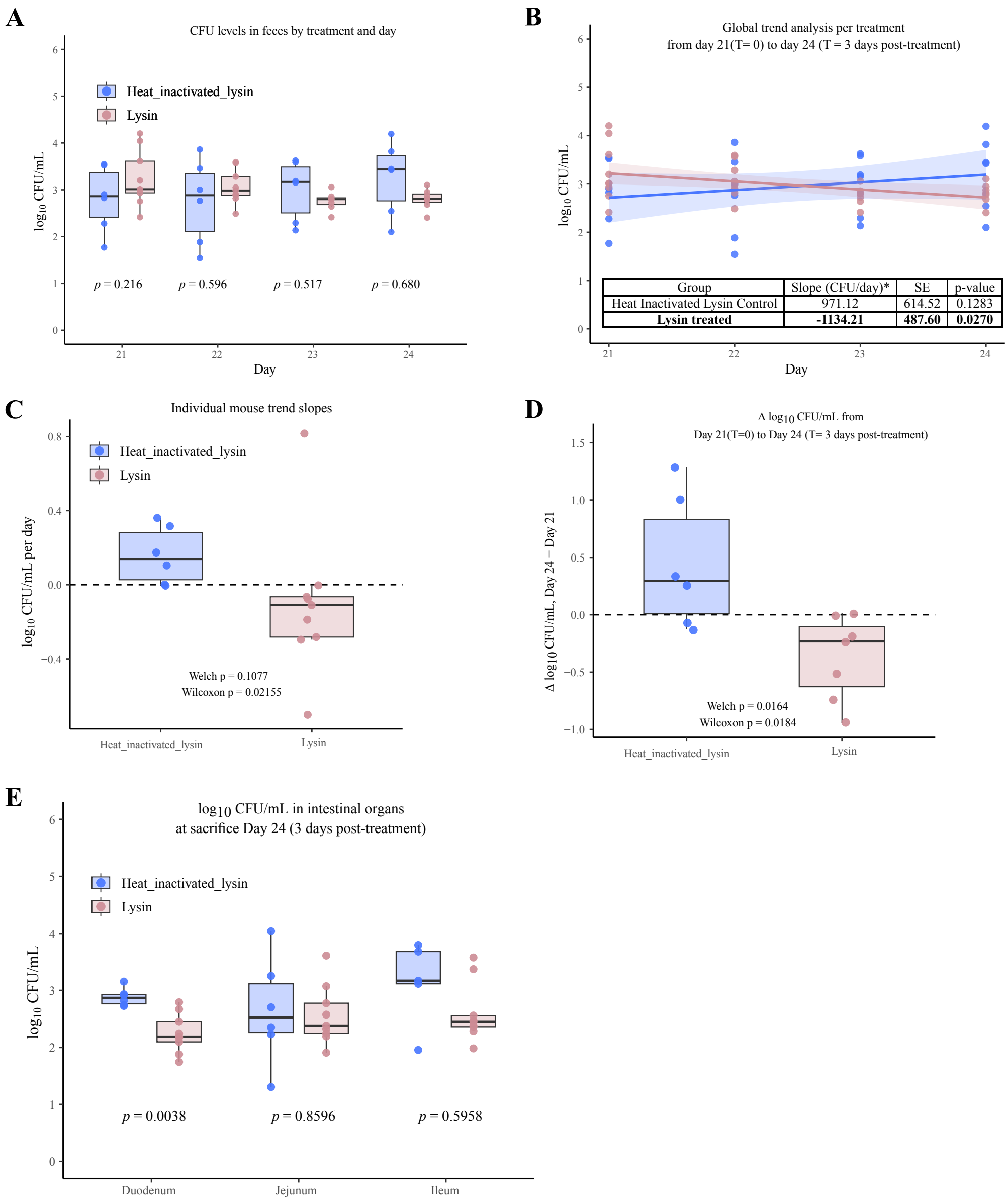

Supplementary Figure 5. Effect of lysin vs heat inactivated lysin treatment on fecal and intestinal CFU levels over time. (A) Fecal bacterial loads expressed as log10 CFU/mL from day 21, corresponding to treatment start, T=0 (day of first gavage), to day 24, corresponding to 3 days post-treatment (3 doses administered), in mice receiving heat-inactivated lysin control or active lysin. Groups were compared at each time point using Mann–Whitney U tests. (B) Global trend analysis of fecal CFU dynamics between day 21 and day 24 for each treatment group. Linear trends are shown for each group, with the corresponding group-level slopes, standard errors, and p-values reported in the table. (C) Individual mouse trajectory slopes calculated from fecal log10 CFU/mL values over time and compared between groups using Welch's t-test and the Mann–Whitney U test. (D) Overall change in fecal bacterial load between day 21 and day 24, expressed as delta log10 CFU/mL, compared between treatment groups using Welch's t-test and the Mann–Whitney U test. (E) Endpoint bacterial loads in intestinal compartments collected at sacrifice on day 24, expressed as log10 CFU/mL and compared between treatment groups for each organ. Boxplots show the distribution of individual mice, with points representing individual animals. The dashed horizontal line in panels C and D indicates no change.

Supplementary Table 1. Strain used for the agar and broth lysin activity assays.

StrainID	Species	Source	Country	Lys_AF151 (Activity agar)	Lys_AF151 (Activity broth)	Cpl-1 (Activity gar)
AF4	*Streptococcus salivarius*	Duodenal	Central African Republic	2	1	0
AF5	*Streptococcus salivarius*	Duodenal	Central African Republic	1	1	0
AF8	*Streptococcus salivarius*	Duodenal	Central African Republic	3	2	0
AF9ms	*Streptococcus salivarius*	Duodenal	Central African Republic	3	3	0
AF10	*Streptococcus salivarius*	Duodenal	Madagascar	2	1	0
AF23	*Streptococcus salivarius*	Duodenal	Central African Republic	2	1	0
AF24	*Streptococcus salivarius*	Duodenal	Central African Republic	2	1	0
AF39	*Streptococcus salivarius*	Duodenal	Central African Republic	1	1	0
AF42	*Streptococcus salivarius*	Duodenal	Central African Republic	1	1	0
AF45	*Streptococcus salivarius*	Duodenal	Central African Republic	1	1	0
AF49	*Streptococcus salivarius*	Duodenal	Central African Republic	3	3	0
AF59	*Streptococcus salivarius*	Duodenal	Central African Republic	3	3	0
AF79	*Streptococcus salivarius*	Duodenal	Central African Republic	3	3	0
AF92	*Streptococcus salivarius*	Duodenal	Central African Republic	3	3	0
AF94	*Streptococcus salivarius*	Duodenal	Central African Republic	1	3	0
AF111ms	*Streptococcus salivarius*	Gastric	Central African Republic	2	3	0
AF112	*Streptococcus salivarius*	Gastric	Central African Republic	3	3	0
AF122	*Streptococcus salivarius*	Gastric	Madagascar	3	3	0
AF127	*Streptococcus salivarius*	Gastric	Central African Republic	2	3	0
AF134	*Streptococcus salivarius*	Gastric	Central African Republic	1	3	0
AF142	*Streptococcus salivarius*	Gastric	Madagascar	1	3	0
AF151	*Streptococcus salivarius*	Gastric	Madagascar	3	3	0
AF155	*Streptococcus salivarius*	Gastric	Madagascar	3	3	0

AFB166	*Streptococcus salivarius*	Stool	Central African Republic	1	2	0
AF177	*Streptococcus salivarius*	Gastric	Central African Republic	0	0	0
AF178	*Streptococcus salivarius*	Gastric	Madagascar	1	2	0
AF187	*Streptococcus salivarius*	Gastric	Madagascar	0	0	0
AF238	*Streptococcus salivarius*	Gastric	Madagascar	3	3	0
AF265	*Streptococcus salivarius*	Gastric	Madagascar	3	3	0
AF276	*Streptococcus salivarius*	Gastric	Madagascar	3	3	0
AF309	*Streptococcus salivarius*	Gastric	Central African Republic	3	3	0
AF316	*Streptococcus salivarius*	Gastric	Madagascar	2	3	0
AF329	*Streptococcus salivarius*	Gastric	Madagascar	2	3	0
AFB347	*Streptococcus salivarius*	Stool	Central African Republic	1	0	0
AF349	*Streptococcus salivarius*	Gastric	Madagascar	3	2	0
AFB466	*Streptococcus salivarius*	Stool	Central African Republic	1	0	0
AFB570	*Streptococcus salivarius*	Stool	Central African Republic	0	0	0
DSM20560	*Streptococcus salivarius*	Blood	NA	3	3	0
HSISS4	*Streptococcus salivarius*	Ileostomy	Netherlands	2	3	0
K12	*Streptococcus salivarius*	Saliva	New Zealand	3	3	0
SAT463	*Streptococcus salivarius*	Stool	Central African Republic	3	NA	0
SAT500	*Streptococcus salivarius*	Stool	Central African Republic	2	NA	0
SAT501	*Streptococcus salivarius*	Stool	Central African Republic	2	NA	0
SAT505	*Streptococcus salivarius*	Stool	Central African Republic	2	NA	0
SAT508	*Streptococcus salivarius*	Stool	Central African Republic	3	NA	0
SAT520	*Streptococcus salivarius*	Stool	Central African Republic	1	NA	0
SAT526	*Streptococcus salivarius*	Stool	Central African Republic	3	NA	0
SAT535	*Streptococcus salivarius*	Stool	Central African Republic	2	NA	0
SAT536	*Streptococcus salivarius*	Stool	Central African Republic	1	NA	0
SAT502	*Streptococcus parasanguinis*	Stool	Central African Republic	0	NA	0

SAT504	*Streptococcus parasanguinis*	Stool	Central African Republic	1	NA	0
SAT506	*Streptococcus parasanguinis*	Stool	Central African Republic	0	NA	0
SAT509	*Streptococcus parasanguinis*	Stool	Central African Republic	1	NA	0
SAT511	*Streptococcus parasanguinis*	Stool	Central African Republic	0	NA	0
SAT529	*Streptococcus parasanguinis*	Saliva	Central African Republic	0	NA	0
SAT532	*Streptococcus parasanguinis*	Saliva	Central African Republic	1	NA	0
SAT551	*Streptococcus parasanguinis*	Saliva	Central African Republic	0	NA	0
SAT561	*Streptococcus parasanguinis*	Saliva	Central African Republic	0	NA	2
SAT565	*Streptococcus parasanguinis*	Saliva	Central African Republic	0	NA	0
SAT521	*Streptococcus mitis*	Saliva	Central African Republic	0	NA	2
SAT522	*Streptococcus mitis*	Saliva	Central African Republic	0	NA	3
SAT523	*Streptococcus mitis*	Saliva	Central African Republic	0	NA	2
SAT524	*Streptococcus mitis*	Saliva	Central African Republic	0	NA	3
SAT527	*Streptococcus mitis*	Saliva	Central African Republic	0	NA	3
SAT534	*Streptococcus mitis*	Saliva	Central African Republic	0	NA	3
SAT538	*Streptococcus mitis*	Saliva	Central African Republic	0	NA	3
SAT563	*Streptococcus mitis*	Saliva	Central African Republic	0	NA	2
SAT568	*Streptococcus mitis*	Saliva	Central African Republic	0	NA	1

Supplementary Table 2. Strain used to generate the spent medium ("donor" strains)

Strain ID	Strain ID2	Source	Bacterial species	Niche overlap
This Study	AFB099	Human feces, Central African Republic	*Coprococcus comes*	YES
This Study	AFB150	Human feces, Central African Republic	*Blautia intestinalis*	YES
This Study	AFB159	Human feces, Central African Republic	*Ruminococcus lactaris*	YES
This Study	AFB180	Human feces, Central African Republic	*Blautia wexlerae*	YES
This Study	AFB204	Human feces, Central African Republic	*Flavonifractor plautii*	YES
This Study	AFB210	Human feces, Central African Republic	*Bacteroides ovatus*	YES

This Study	AFB226	Human feces, Central African Republic	*Coprococcus eutactus*	YES
This Study	AFB290	Human feces, Central African Republic	*Blautia glucerasea*	YES
This Study	AFB426	Human feces, Central African Republic	*Ruminococcus bromii*	YES
This Study	AFB475	Human feces, Central African Republic	*Blautia luti*	YES
This Study	AFB478	Human feces, Central African Republic	*Blautia caecimuris*	YES
This Study	AFB481	Human feces, Central African Republic	*Paraclostridium bifermentans*	YES
This Study	AFB493	Human feces, Central African Republic	*Blautia obeum*	YES
This Study	AFB594	Human feces, Central African Republic	*Odoribacter splanchnicus*	YES
This Study	EBT030	Human feces, Switzerland	*Bacteroides intestinalis*	YES
This Study	EBT048	Human feces, Switzerland	*Bacteroides faecis*	NO
This Study	EBT058	Human feces, Switzerland	*Bacteroides caccae*	YES
This Study	EBT067	Human feces, Switzerland	*Mediterraneibacter faecis*	YES
This Study	EBT072	Human feces, Switzerland	*Anaerobutyricum hallii*	NO
This Study	EBT151	Human feces, Switzerland	*Anaerobutyricum hallii*	NO
This Study	EBT260	Human feces, Switzerland	*Ruminococcus faecis*	YES
DSM3319	PV008	Human feces	*Anaerostipes hadrus*	YES
CCUG69734	PV015	Human blood, Sweden	*Anaerotruncus colihominis*	YES
ATCC29236	PV025	NA	*Blautia coccoides*	YES
DSM27629	PV027	Human feces, Republic of Korea	*Blautia faecis*	YES
DSM20583	PV037	Human feces	*Blautia hansenii*	YES
DSM10518	PV067	Rumen of suckling lamb, France	*Blautia schinkii*	YES
DSM105336	PV086	Wild boar, Canada	*Lacrimispora celerecrescens*	YES
ATCC25537	PV096	Calf rumen	*Enterocloster clostridioformis*	YES
DSM108250	PV111	Human feces, Austria	*Clostridium symbiosum*	YES
ATCC27761	PV119	NA	*Coprococcus catus*	YES
DSM15684	PV158	Human feces, USA	*Eubacterium ramulus*	YES
DSM108070	PV200	Human feces, Scotland	*Roseburia inulinivorans*	NO

Supplementary Tables 3. Mice related measurement and statistics with PBS control

Raw CFU/mL in feces

MouseID	Treatment	Day 21	Day D22	Day 23	Day 24
XB_0119	+ Lysin	1027.7	653.3	258.2	602.2
XB_0120	+ Lysin	1574.1	965.1	436.8	482.5
XB_0121	PBS control	402.7	1131.3	615.3	571.4
XB_0122	PBS control	937.2	334.9	167.3	514.3
XB_0123	PBS control	2040.2	5154.7	1963.0	908.6
XB_0124	PBS control	2125.6	2544.1	1065.2	1039.7
XB_0125	+ Lysin	859.4	756.9	634.0	889.0
XB_0126	+ Lysin	4091.6	3915.6	713.4	742.8
XB_0127	+ Lysin	259.2	1907.9	160.7	254.0
XB_0128	+ Lysin	1021.2	802.8	530.5	647.8
XB_0129	PBS control	3599.9	1818.7	4200.6	3073.2
XB_0130	PBS control	1366.9	1203.8	1831.6	2693.4

Raw CFU/mL in intestinal organs

MouseID	Treatment	Duodenum	Jejunum	Ileum
XB_0119	+ Lysin	75.8	597.0	242.4
XB_0120	+ Lysin	154.4	176.6	194.2
XB_0121	PBS control	295.9	230.3	41.1
XB_0122	PBS control	127.3	422.8	258.3
XB_0123	PBS control	197.6	270.0	2399.1
XB_0124	PBS control	177.4	236.6	796.3
XB_0125	+ Lysin	55.6	241.9	231.3
XB_0126	+ Lysin	287.2	211.9	297.5
XB_0127	+ Lysin	176.6	155.8	96.1
XB_0128	+ Lysin	468.3	80.8	362.7
XB_0129	PBS control	267.5	673.8	608.9
XB_0130	PBS control	181.5	2214.7	669.0

CFU/mL in feces of mice treated with lysin or PBS control

Day	Control (mean ± SD)	Lysin (mean ± SD)	p-value
21	1745.4 ± 1119.8	1472.2 ± 1350.5	0.589
22	2031.2 ± 1699.2	1500.3 ± 1268.3	0.485
23	1640.5 ± 1431.6	455.6 ± 214.6	0.093

24	1466.8 ± 1121.4	603.1 ± 218.9	0.132

*Values are mean ± SD. p-values were calculated using Mann–Whitney U test. (N=6).

CFU/mL in small intestinal segments of mice treated with lysin or PBS control

Organ	Control (mean ± SD)	Lysin (mean ± SD)	p-value
Duodenum	207.9 ± 62.5	203.0 ± 153.9	0.093
Ileum	795.4 ± 834.3	237.4 ± 90.8	0.132
Jejunum	674.7 ± 773.1	244.0 ± 181.4	0.065

*Values are mean ± SD. p-values were calculated using Mann–Whitney U test. (N=6).

Trend analyses of fecal CFU counts with lysin or PBS control

Group	Slope (CFU/day)*	SE	p-value
PBS control	-122.7	239.3	0.613
**Lysin treated**	**-365.2**	**170.5**	**0.044**

* Linear regression was applied to replicate-level data across days (D21–D24). The slope indicates the estimated daily change in CFU/ml. A negative slope indicates a downward trend. Trend analysis was performed using linear regression, with time modeled as a continuous variable. Trend analysis was performed using linear regression, with time modeled as a continuous variable. The p-value corresponds to the t-test of the regression coefficient for time, assessing whether the slope differed from zero.

Mouse body weight (g) at different days post-arrival with lysin or PBS control

MouseID	Treatment	Day0	Day7	Day14	Day21	Day22	Day23	Day24
XB_0119	+ Lysin	10.2	11.6	12.6	14.0	14.6	14.7	14.7
XB_0120	+ Lysin	10.4	11.9	13.4	15.2	15.5	15.9	16.0
XB_0121	PBS control	10.4	12.0	13.0	14.3	14.8	14.9	14.8
XB_0122	PBS control	9.6	11.2	12.2	14.3	13.7	13.5	13.8
XB_0123	PBS control	10.2	10.8	12.2	14.0	14.0	14.0	14.3
XB_0124	PBS control	9.9	11.7	12.3	13.2	14.1	14.4	14.3
XB_0125	+ Lysin	9.6	10.9	12.5	14.0	14.3	14.5	14.0
XB_0126	+ Lysin	9.7	12.1	13.2	14.9	15.2	15.0	14.9
XB_0127	+ Lysin	9.7	11.9	13.1	14.6	15.0	14.9	15.1
XB_0128	+ Lysin	10.4	11.7	13.6	14.6	15.1	15.5	15.6
XB_0129	PBS control	9.9	12.0	13.9	15.9	16.1	16.6	17.1
XB_0130	PBS control	9.6	11.7	13.8	15.8	16.3	16.6	16.9
Mean ± SD (Control vs Lysin)		9.93 ± 0.32 vs 10.00 ±	11.57 ± 0.48 vs 11.68 ±	12.90 ± 0.79 vs 13.07 ±	14.58 ± 1.06 vs 14.55 ±	14.83 ± 1.12 vs 14.95 ±	15.00 ± 1.32 vs 15.08 ±	15.20 ± 1.43 vs 15.05 ±

		0.37 (p=0.744)	0.42 (p=0.808)	0.44 (p=0.575)	0.48 (p=0.936)	0.43 (p=0.589)	0.52 (p=0.630)	0.70 (p=0.810)

Note: Values are presented as individual mouse measurements and group means ± SD. p-values are from Mann–Whitney U tests (two-sided).

Mouse body weight gain vs Day 0 (g) with lysin or PBS control

MouseID	Treatment	Gain at D21	Gain at D22	Gain at D23	Gain at D24
XB_0119	+ Lysin	3.8	4.4	4.5	4.5
XB_0120	+ Lysin	4.8	5.1	5.5	5.6
XB_0121	PBS control	3.9	4.4	4.5	4.4
XB_0122	PBS control	4.7	4.1	3.9	4.2
XB_0123	PBS control	3.8	3.8	3.8	4.1
XB_0124	PBS control	3.3	4.2	4.5	4.4
XB_0125	+ Lysin	4.4	4.7	4.9	4.4
XB_0126	+ Lysin	5.2	5.5	5.3	5.2
XB_0127	+ Lysin	4.9	5.3	5.2	5.4
XB_0128	+ Lysin	4.2	4.7	5.1	5.2
XB_0129	PBS control	6.0	6.2	6.7	7.2
XB_0130	PBS control	6.2	6.7	7.0	7.3
Mean ± SD (Control vs Lysin)		4.65 ± 1.21 vs 4.55 ± 0.51 (p=0.873)	4.90 ± 1.23 vs 4.95 ± 0.42 (p=0.422)	5.07 ± 1.42 vs 5.08 ± 0.35 (p=0.468)	5.27 ± 1.54 vs 5.05 ± 0.49 (p=0.467)

Note: Values are presented as individual mouse measurements and group means ± SD. p-values are from Mann–Whitney U tests (two-sided).

Supplementary Tables 4. Mice related measurement and statistics with heat inactivated lysin

Raw CFU/mL in feces of mice treated with lysin or heat inactivated lysin control

MouseID	Treatment	Day 21	Day D22	Day 23	Day 24
XB_0161	Heat inactivated lysin Control	3327.4	1005.2	1532.2	2786.1
XB_0162	Heat inactivated lysin Control	58.7	34.9	195.4	125.3
XB_0163	Heat inactivated lysin Control	190.0	76.5	136.3	349.0
XB_0164	Heat inactivated lysin Control	797.8	7269.0	3851.0	15633.9
XB_0165	Heat inactivated lysin Control	3532.7	581.9	1415.5	2651.3
XB_0166	Heat inactivated lysin Control	665.4	2842.6	4222.0	6601.4
XB_0167	+ Lysin	15954.9	307.0	629.1	8537.0
XB_0168	+ Lysin	565.8	3708.1	6013.0	9171.8
XB_0169	+ Lysin	11117.8	1120.2	1138.8	1265.1

Raw CFU/mL in intestinal organs of mice treated with lysin or heat inactivated lysin control

MouseID	Treatment	Duodenum	Jejunum	Ileum
XB_0161	Heat inactivated lysin Control	822.6	1795.3	0.6
XB_0162	Heat inactivated lysin Control	855.9	505.4	1306.5
XB_0163	Heat inactivated lysin Control	664.9	20.2	90.2
XB_0164	Heat inactivated lysin Control	535.1	11089.3	6257.5
XB_0165	Heat inactivated lysin Control	1427.1	226.4	1479.8
XB_0166	Heat inactivated lysin Control	557.1	170.4	4804.7
XB_0167	+ Lysin	149.0	376.7	2362.1
XB_0168	+ Lysin	619.0	4082.0	3780.2
XB_0169	+ Lysin	125.1	1185.2	286.1

CFU/mL in feces of mice treated with lysin or heat inactivated lysin control

Day	Control (mean ± SD)	Lysin (mean ± SD)	p-value
21	1745.4 ± 1119.8	1472.2 ± 1350.5	0.216
22	2031.2 ± 1699.2	1500.3 ± 1268.3	0.596
23	1640.5 ± 1431.6	455.6 ± 214.6	0.517
24	1466.8 ± 1121.4	603.1 ± 218.9	0.680

*Values are mean ± SD. p-values were calculated using Mann–Whitney U test. (N=6).

CFU/mL in small intestinal segments of mice treated with lysin or heat inactivated lysin control

Organ	Control (mean ± SD)	Lysin (mean ± SD)	p-value
**Duodenum**	**810,45 ± 329,75**	**234,56 ± 190,89**	**0.0038**

Ileum	2323,22 ± 2598,55	872,51 ± 1298,03	0.5958
Jejunum	2301,17 ± 4353,38	789,77 ± 1280,51	0.8596

*Values are mean ± SD. p-values were calculated using Mann–Whitney U test. (N=6).

Trend analyses of fecal CFU counts

Group	Slope (CFU/day)*	SE	p-value
Heat Inactivated Lysin Control	971.12	614.52	0.1283
**Lysin treated**	**-1134.21**	**487.60**	**0.0270**

* Linear regression was applied to replicate-level data across days (D21–D24). The slope indicates the estimated daily change in CFU/ml. A negative slope indicates a downward trend. Trend analysis was performed using linear regression, with time modeled as a continuous variable. Trend analysis was performed using linear regression, with time modeled as a continuous variable. The p-value corresponds to the t-test of the regression coefficient for time, assessing whether the slope differed from zero.

Mouse body weight (g) at different days post-arrival with lysin or heat inactivated lysin control

MouseID	Treatment	Day0	Day7	Day14	Day21	Day22	Day23	Day24
XB_0161	HILC	9.1	11.3	12	13.9	14.2	14.9	14.6
XB_0162	HILC	10.5	12.2	12.9	14.3	14.6	15.1	14.8
XB_0163	HILC	10	12.2	13.4	15.8	15.5	15.6	15.4
XB_0164	HILC	10.3	12.1	13.2	15.1	15.2	15.5	16.1
XB_0165	HILC	10	12	12	13.4	14	14	14.2
XB_0166	HILC	10	12	12.6	14.5	13.7	14.6	14.9
XB_0167	+ Lysin	10.2	12.5	13.4	14.7	15.1	15.3	15.6
XB_0168	+ Lysin	10	12.1	13.1	14.4	14.5	14.9	15.1
XB_0169	+ Lysin	10	12.1	12.7	14.6	15	14.7	15.1
Mean ± SD (Control vs Lysin)		9.98 ± 0.48 vs 10.07 ± 0.12 (p=1.00)	11.97 ± 0.34 vs 12.23 ± 0.23 (p=0.35)	12.68 ± 0.59 vs 13.07 ± 0.35 (p=0.44)	14.50 ± 0.86 vs 14.57 ± 0.15 (p=0.71)	14.95 ± 0.60 vs 14.97 ± 0.31 (p=1.00)	15.00 ± 0.67 vs 15.27 ± 0.29 (p=0.36)	15.20 ± 1.43 vs 15.05 ± 0.70 (p=0.81)

Note: Values are presented as individual mouse measurements and group means ± SD. p-values are from Mann–Whitney U tests (two-sided). HILC = Heat inactivated lysin control.

Mouse body weight gain vs Day 0 (g) with lysin or heat inactivated lysin control

MouseID	Treatment	Gain at D21	Gain at D22	Gain at D23	Gain at D24
XB_0161	HILC	4.8	5.1	5.8	5.5

XB_0162	HILC	3.8	4.1	4.6	4.3
XB_0163	HILC	5.8	5.5	5.6	5.4
XB_0164	HILC	4.8	4.9	5.2	5.8
XB_0165	HILC	3.4	4	4	4.2
XB_0166	HILC	4.5	3.7	4.6	4.9
XB_0167	+ Lysin	4.5	4.9	5.1	5.4
XB_0168	+ Lysin	4.4	4.5	4.9	5.1
XB_0169	+ Lysin	4.6	5	4.7	5.1
Mean ± SD (Control vs Lysin)		4.52 ± 0.85 vs 4.50 ± 0.10 (p=1.000)	4.55 ± 0.70 vs 4.80 ± 0.26 (p=0.714)	4.97 ± 0.67 vs 4.90 ± 0.20 (p=1.000)	5.02 ± 0.64 vs 5.20 ± 0.17 (p=0.714)

Note: Values are presented as individual mouse measurements and group means ± SD. p-values are from Mann–Whitney U tests (two-sided). HILC = Heat inactivated lysin control.
